# Cerebro-Spinal Fever

**Published:** 1919-04-12

**Authors:** 


### CEREBRO-SPINAL FEVER.

### A Triumph for Medical Investigation.

In some quarters there have of late appeared complaints regarding a sporadic outbreak of "spotted fever," but it is certain that the originators were but little cognisant of the real extent of medical advance and victory over this disease during the past few years. At one time Europe, Northern Africa, and America were threatened, and it is now no secret that for a few months the position appeared serious in the extreme. Organisation and energy then saved the situation, and to-day the combined results place the bacteriologist and the public-health authority in a position satisfactorily to control the disease. The effort began with the establishment of a central laboratory in London for special instruction of the army of workers needed. University, Service, and school laboratories were requisitioned, and even a fast motor bacteriological van was equipped for immediate removal to the scene of an outbreak. A complete carrier "intelligence" department was evolved, and every possible step taken to co-ordinate its work with that of the laboratories. What one may term the outside and executive movements became both widespread and efficient, but the key to final success lay in research work upon the exact characters of the causal organism.

### The Meningococcus.

In 1914, although a number of somewhat inconclusive experiments upon the causal organism of "spotted fever" had been commenced, our knowledge was really limited to the fact that it belonged to the great class of gram-negative diplococci in practice, differentiated more by the clinic symptoms it produced than by morphological and cultural characteristics. In fact, many held that the germ of Weichselbaum was not a distinct species, but a stage in which other common members of the class might at times appear, or that it was a "pleomorphic" organism. The enormous amount of investigation called in action when the serious possibilities were realised resulted, within little more than twelve months, in the isolation of a true meningococcus, or at least of a definite species of causal germ. By methods of agglutination there are found two main and two auxiliary races, but most observers now agree that the division is to some extent artificial, and that one may now safely describe the general characters of a specific meningococcus.

By many the character of the plated colonies is considered so typical that in it the chief reliance for recognition is placed. Importance is laid upon the small size, the translucency and sharp definition of the colonies, and upon the development of a small central granular area. A forty-eight-hour culture will show all these points, but as it is unsafe to delay sub-culture so long, the custom is to pick off suspicious colonies at twenty-four hours, and re-observe in a fresh media plate. Most strains of meningococcus fail to grow at 23° C., whereas most of the similar *g*-negative organisms of the naso-pharynx multiply more or less readily at this temperature. The coccus forms acid in glucose and maltose, but not from galaclose, saccharose, or lactose. Singularly intolerant of drying, which kills it in two to five minutes, it will stand exposure to sunlight for several hours. Such is a rough outline of the general properties of the organism, but the secret of these observations has been in its laboratory isolation, in the invention of new culture media.

### Cultural Characters.

The meningococcus has long been notoriously difficult to grow, and research in the past has been greatly hindered by media difficulties. The nutrose (used in nutrose-ascitic-agar) and Witte's peptone formerly used became unprocurable early in the war, and substitutes tried proved unsatisfactory until legumin-agar, or "Trypagar" was introduced. With this medium, a higher positive growth was obtained and by adding 2 per cent. normal horse serum or ascitic fluid not only was the size of colonies increased, but also their vitality under ordinary laboratory conditions prolonged.

### Natural Occurrence in Naso-Pharynx.

In connection with the natural conditions under which the germ may exist or survive in the human body, some interesting points have been decided. Not only in culture are pneumococci and streptococci found to be strongly antagonistic to the growth of a meningococcus, but also in any natural site. The saliva, normally containing examples of streptococci, is markedly inimical, but with the naso-pharyngeal secretion which in non-catarrhal subjects is relatively free from these organisms, and is in fact, strongly antagonistic to them, the meningococcus is actually aided in its development. An example is seen in the frequent disappearance of the cerebra-spinal fever germ in carrier cases at the onset of a severe cold in the head and similar points relating to the ease with which the common cocci of inflammation and catarrh destroy a meningococcus implanted with them explain many of the difficulties experienced in carrier detection and emphasise the necessity for extraordinary care in avoidance of swab contamination in collecting specimens.

### Diagnostic Procedures.

Apart from examination of the nasal passages, the next most important is the diagnostic evidence, obtained from the cerebro-spinal fluid. By most observers, the mere finding of gram-negative cocci in this fluid is now held to be conclusive. There are a very few instances where the cultivation of another and non-menigeal member of the group has been claimed, but the evidence is not well substantiated, and the real point of weakness in this direc-

**Cerebro-Spinal Fever—(*continued*).**

tion lies in the occasional failure to produce any artificial growth whatever from the fluid of clinically typical cases. Several reasons may, however, be assigned for this. In fulminating cases, the coccus may not have had time to appear, and overwhelming septicæmia causing death before the meningeal characteristics can develop. In very mild cases where rapid recovery occurs, in instances where the puncture is performed very early in the disease, or in cases become chronic where the germ is localised to the meningeo, the fluid may give negative results. The technique of naso-pharyngeal detection has, however, been so greatly improved that diagnosis can be made with a high percentage of certainty and, combined with the lumbar puncture result, provides a good foundation for certainty.

### Antimenigococcal Sera.

After any methods of isolation, the turning-point in both treatment and diagnosis has been the production of efficient agglutinating sera. Details of direct and indirect agglutination tests are too extensive to be recorded here, but it may be stated that after the most patient work, specific anti-sera for each sub-species of meningococci have been produced. Major Gordon, R.A.M.C., draws the conclusion from experimental results that, under laboratory and epidemic conditions alike, four serological types of meningococci breed true and that the recent outbreaks of disease have been due, not to transient unstable variants of a single organism, but to a group of individual species of meningococcus of which the first two between them have so far accounted for over 80 per cent. of the cases. The third and fourth account for practically the whole of the remaining 20 per cent., the exceptions being sufficiently rare to be here left with the mere reference. To these four types, specific proved anti-sera have been prepared.

### The Carrier Condition.

Convincing evidence has been obtained that the main factor in the spread of disease is the healthy carrier who harbours the meningococcus in his naso-pharynx. The mode of droplet infection, masks, etc., has been too recently under consideration in influenza cases to call for repetition, but the extent to which the carrier himself is infested, his sociability, and the degree of bad ventilation or crowding, in his immediate environment must be remembered as having an important influence on the spread. In all outbreaks carriers have greatly outnumbered the cases. Usually if no active symptoms develop they become free in a relatively short time under normal hygienic conditions, but there invariably remain a few who become chronic and undoubtedly constitute the source of either outbreaks or sporadic cases of cerebro-spinal fever. Antiseptic treatment by usual means has been singularly ineffective with these carriers, and may be explained by the fact that the antiseptic did not reach all the areas showing meningococci, and also that the organisms are frequently found in the depth of the mucous membrane. At present the best way out of this difficulty has been the use of atomisers which in an enclosed space distribute throughout the atmosphere the minutest particles of disinfectant, and so reach the human mucous membrane in a way exactly analogous to the original mode of infection. A solution of one to two per cent. chloramine-T (Dakin) or one to two per cent. zinc sulphate have been found the most efficient for general use, and in a room of 750 cubic feet a litre of such solution is sprayed in the course of fifteen to twenty minutes, during the whole of which the carriers remain in the room and inhale as much as possible through the nose. It is suggested that too fine a spray is not advisable, as the droplets may not in that case be arrested in the naso-pharynx but enter in great number the bronchial tubes, setting up considerable irritation. On the whole, however, the method has proved extremely satisfactory for troops and ships' companies, and with improvement in technical details has become an efficient method of clearing the carrier danger.

### Treatment by Anti-Serum.

As explained earlier, the reasons for the pre-war failure and low estimate of the value of serum treatment have been, first, weak strength of artificially prepared sera against any type of meningococci and secondly, the failure to appreciate the four distinct sub-species of the germ. With improved technique, both polyvalent and type anti-sera have now been produced, and the modern treatment scheme is to inject polyvalent serum via lumber puncture at the first suspicion of disease and to then attempt immediate recognition of the exact type of organism, determine its agglutinating power and continue later injections with the appropriate type anti-serum. This procedure can now be carried out sufficiently quickly to make the method absolutely specific and to reduce the mortality rate in a remarkable degree. Service records give as low an estimate as eighteen per cent. for acute cases.

### Conclusion.

The history of this medical triumph is not one of discovery but rather of painstaking, enterprising investigation of previously accepted facts: a realisation that pre-war explanation could not satisfactorily account for the disease as actually encountered, and a whole-hearted attempt to polish and refine our understanding of its variations.

The organisms and its sub-species can be recognised and grown with considerable certainty, it is possible to prepare a high titre anti-serum, capable of being produced and stored on a large scale, and the carrier question is as well in hand as with the majority of infectious diseases. A few years ago our preventive means against this infection were slender in the extreme. To-day, after many actual and many threatened outbreaks, we stand in a position which, with but little more confirmatory evidence, will place cerebro-spinal fever in the increasing list of defeated diseases.

